# Do High Levels of Maternally Derived Antibodies Interfere with the Vaccination of Piglets against Porcine Circovirus Type 2? A Literature Review and Data Analysis

**DOI:** 10.3390/vaccines9080923

**Published:** 2021-08-19

**Authors:** Barbara Poulsen Nautrup, Ilse Van Vlaenderen, Choewkong Mah, Jose Angulo

**Affiliations:** 1 EAH-Consulting, 52064 Aachen, Germany; 2 CHESS, 2820 Bonheiden, Belgium; ivanvlaenderen@chessinhealth.com; 3 Zoetis, Parsippany, NJ 07054, USA; ck.mah@zoetis.com (C.M.); Jose.Angulo@zoetis.com (J.A.)

**Keywords:** porcine circovirus type 2, maternally derived antibodies, vaccination, production parameters, viremia, immunological parameters

## Abstract

Vaccination against porcine circovirus type 2 (PCV2) is commonly performed in piglets worldwide, and increasingly also in sows. We conducted a literature search and review to assess the potential interference of maternally derived antibodies (MDA) in piglets with vaccination against PCV2. The effectiveness of vaccination was compared to no vaccination in the presence of high levels of MDA (≥8 log_2_ IPMA titer), as reported in field studies. In total, 13 papers fulfilled the predefined inclusion criteria, allowing up to 24 comparisons per parameter. In the presence of high levels of MDA, vaccinated pigs had, on average, a 20 g/d higher mean daily weight gain and a 34% lower mortality compared to non-vaccinates. The maximum percentage of viremic pigs was reduced by 63% and the maximum viral load in serum was 0.72 log_10_ PCV2 DNA copies lower. Vaccination at 3 weeks of age was associated with the highest improvements in production parameters and reductions in viremia. Our findings suggest that the vaccination of piglets is effective with respect to production parameters and viremia even in the presence of high MDA, with an age of 3 weeks at vaccination being most beneficial.

## 1. Introduction

Porcine circovirus type 2 (PCV2) is recognized as one of the most important pathogens of the pig population worldwide [1]. PCV2 causes a disease complex previously described as post-weaning multisystem wasting syndrome, which is grouped together with many other PCV2-associated clinical conditions (such as respiratory syndrome, enteritic disease, dermatitis and nephropathy syndrome as well as reproductive failure) as porcine circovirus-associated disease (PCVAD) [2]. The clinical manifestations of PCVAD vary from subclinical infections to severe, deadly porcine circovirus-associated disease. PCVAD may manifest as a sporadic individual animal diagnosis, but also as severe herd problem [1].

The first commercial PCV2 vaccines became available in 2004 in Europe and 2006 in North America, leading to a decrease in morbidity and improved production, thereby becoming the single best-selling prophylactics in porcine husbandry [3]. Consequently, the use of vaccination has shifted the impact of PCV2 on general pig health from a period of worldwide severe clinical outbreaks (1997–2007) to self-limiting subclinical infections with occasional outbreaks. With the widespread vaccination of piglets and the increasing use of PCV2 vaccines in sows, levels of maternally derived antibodies (MDA) and their potential interference with the effectiveness of piglet vaccination have become increasingly important [4].

Potential MDA interference has been studied at different levels, such as the impact on immunological markers or clinical and productive parameters. In terms of the vaccine-elicited humoral immune response, it has been shown that high MDA levels at the time of vaccination jeopardize vaccine-induced seroconversion in piglets [5,6]. The effect of MDA on average daily weight gain (ADWG), however, was assessed differently in various studies [7,8]. The impact of vaccination seems to be dependent on the level of MDA at the time of vaccination [7] and the age at vaccination. Pigs vaccinated at 3 weeks of age had higher ADWG than piglets vaccinated at 1 week of age if MDA levels were high at the time of immunization [8]. Accordingly, the overall impact of MDA on the effectiveness of vaccination seems to be dependent on various factors and can hardly be determined from single studies.

This study aimed to evaluate the potential interference of MDA on the vaccination of piglets against PCV2, as reported in field studies identified by the literature review, thereby also investigating the effect of potential co-variates.

## 2. Materials and Methods

### 2.1. Research Questions and General Approach

The following two research questions were defined a priori, to be answered from published studies as identified from literature review: (1) Is the vaccination of piglets against PCV2 effective in the presence of high levels of MDA (primary analysis)? (2) Does the effectiveness of vaccination differ in cases of high versus low to moderate levels of MDA (secondary analysis)? In order to address the first research question, outcomes in vaccinated and unvaccinated piglets with high levels of MDA at the age of vaccination were compared. From the studies included to address research question 1, a subgroup of studies which also reported results for piglets with low to moderate levels of MDA at vaccination was evaluated to address research question 2.

The following parameters were defined as outcomes of interest: ADWG and mortality (both over the entire study observation period), maximum percentage of viremic pigs and maximum PCV2 DNA copies in serum (both as measured at any time point during the study observation period), as well as the minimum titer of antibodies (ABs) measured at any time point during the study observation period. As potential covariates, the following parameters were considered: (a) the type of vaccine(s) used for piglet vaccination (inactivated chimeric PCV1-2, inactivated subunit ORF2, or inactivated PCV2 whole virus vaccine); (b) the number of doses and age at dose administration, categorized as follows: 1 dose at <3 weeks of age; 1 dose at 3 weeks of age; 1 dose at >3 weeks of age; 2 doses (with both dates of vaccination being recorded); (c) the level of MDA at vaccination (between 8 and 10 log_2_ or ≥10 log_2_ IPMA titer); and (d) the occurrence of vaccine-induced seroconversion (yes/no), defined as an increase in AB titer between 2 and 6 weeks after vaccination not observed in unvaccinated pigs.

### 2.2. Semi-Systematic Review and Data Collection

The search was conducted as a structured (semi-systematic) review. MEDLINE and the CABI databases were searched on 24–28 September 2020 and timeliness was rechecked on 20 November 2020. Additionally, bibliographies of relevant manuscripts were also hand-searched. The search strategy using MEDLINE and CABI databases included the following search terms: (porcine circovirus type 2 OR PCV2) AND ((vaccination OR vaccine) AND (pig OR piglet)) AND antibody.

The first search level evaluated study eligibility by screening titles and/or abstracts. Eligibility criteria included: (a) natural, i.e., environmental infection (no experimental challenge studies); (b) studies comparing at least two groups of piglets, one group being vaccinated against PCV2 and one group not being vaccinated (control group); (c) the presence of MDA at the time of vaccination that could be due to vaccination or natural infection of sows and that had to be reported as “high level” or level not defined (i.e., subject to full-text evaluation); and (d) the availability of full texts in English, German or Spanish languages. We considered only full papers (no proceeding papers) which had been published within the last 15 years (2005 until the day of last search). Study location was not restricted to any region. The first 50 hits retrieved from the CABI database were assessed by the 1st and 2nd reviewers (the assignment of authors to 1st and 2nd reviewer is given in the Author Contributions). After discussion and agreement, the screening was finalized by the 1st reviewer.

All eligible papers were subject to full-text review (second search level), where the following 2 inclusion criteria applied: (a) the presence of high levels of MDA (PCV2-specific AB) in the serum of vaccinated and unvaccinated piglets around the time of vaccination, being defined as ≥8 log_2_ IPMA titer (if the MDA titer was reported as ELISA S/P or O/D ratio or ELISA titer, the existence of a rational conversion rate for the used ELISA to log_2_ IPMA titer was mandatory); and (b) reporting of at least one of the a priori defined outcome parameters together with minimum statistics required for analysis. The use of a non-commercial, i.e., experimental vaccine and inconsistencies that seriously questioned the outcomes served as exclusion criteria. This second search level was performed by the 1st reviewer, and the 2nd reviewer reassessed 50% of the excluded studies for the appropriateness of exclusion.

From the included papers, all available outcome parameters and co-variates were recorded in an Excel sheet (third search level) that had been developed in advance. This task was performed by the 1st reviewer, and the 2nd reviewer reassessed 25% of the included studies on the appropriateness/completeness of data extraction.

In case of discrepancy between the 2 reviewers at any search level, results were discussed, and corrective measures agreed upon if appropriate.

Most results from serological and viral DNA analyses were depicted only in line or bar charts rather than reported as actual values. In these cases, mean values and standard deviations or standard errors were approximated from the graphs using commercial software developed for digitizing data from scanned graphs (Digitizelt, version 2.3.3; Braunschweig, Germany).

### 2.3. Data Analysis

For the primary analysis (comparison of vaccinated and unvaccinated pigs with high levels of MDA at the age of vaccination), overall analyses were run, including all available comparisons for each parameter. Additionally, subgroup analyses were used to evaluate the potential impact of the defined co-variates on the outcomes. For the secondary analysis (comparing vaccinated pigs with high versus low to medium levels of MDA at vaccination), overall analyses were run for all parameters except minimum AB titers, as the AB titer in the low to medium MDA group was, by definition, lower than in the high MDA group. No subgroup analyses were performed because of the low number of studies included.

Data were analyzed using the statistical software CMA version 2.2 (Biostat, Englewood, NJ, USA). Effect size was the weighted mean difference (ADWG, maximum PCV2 DNA virus copies and minimum AB titer in serum) or the risk ratio (percentage mortality and maximum percentage of viremic piglets). Studies were weighted by variance. For the overall analyses, a random-effects model was used. *p*-values (test of null) were estimated using the Z-statistics. In case that no events or 100% events were recorded in both groups for the binary parameters (mortality and maximum percentage of viremic pigs), the respective study was omitted for that parameter, as recommended by the Cochrane Collaboration, because it did not provide information about the relative probability [9].

For subgroup analyses, the mixed-effects model was used, as commonly recommended in meta-analyses [10]. Heterogeneity between subgroups was estimated using Q-statistics, thereby evaluating whether differences in outcomes could be explained by the respective co-variates. Statistical significance was declared based on two-tailed tests at *p* < 0.05.

A potential publication or selection bias was estimated using the classic fail-safe N approach, which estimates the number of additional hypothetical studies with zero effect required to make the *p*-value for the summary effect no longer significant [11]. The test was run for the ADWG dataset (primary analysis).

## 3. Results

### 3.1. Systematic Literature Search

**Figure 1:**
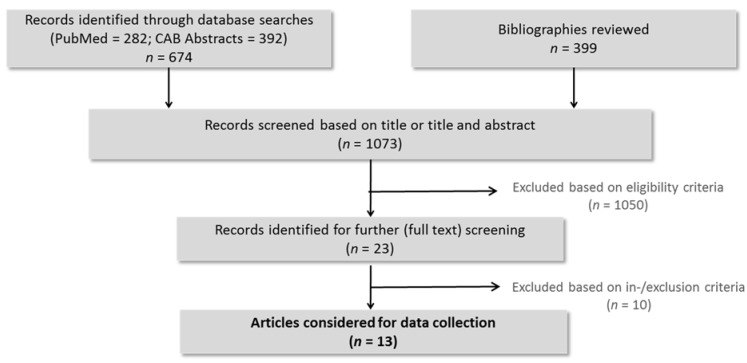
Flow chart of the study selection process, illustrating the study selection process according to PRISMA (Preferred Reporting Items for Systematic Reviews and Meta-Analysis).

The search strategy revealed 674 papers from MEDLINE and the CABI databases. Combined with 399 papers listed in the bibliographies, a total of 1073 papers were screened on the title and/or abstract. Twenty-three records fulfilled the eligibility criteria, thus being subject to full text review. From these papers, 10 were excluded based on in-/exclusion criteria. Accordingly, 13 articles [4,5,7,8,12,13,14,15,16,17,18,19,20] were finally considered for data collection (Figure 1).

Five papers reported outcomes for more than one study group (with a maximum of six individual study groups per paper). Most studies were conducted in Europe, except three which were carried out in Colombia, Mexico, and Thailand. The inactivated subunit vaccines containing baculovirus and expressing PCV2-ORF 2 protein were used most often, whereas inactivated vaccines containing chimeric PCV1-2 or PCV2 whole viruses were used in one and two comparisons each. Table 1 presents an overview of all papers, the main study characteristics (defined as co-variates), as well as the number of comparisons which were eligible for each parameter of interest.

Three of the included papers additionally reported outcomes for pigs with low to moderate levels of MDA at vaccination, thus being eligible for secondary analysis (comparison of vaccinated pigs with high versus low to medium levels of MDA). One of these articles enabled four comparisons for each production parameter (ADWG and mortality). The main study characteristics as well as the number of comparisons which were eligible for each parameter of interest are given in Table 2.

### 3.2. Data Analyses

#### 3.2.1. Primary Analysis (Vaccinated Pigs vs. Unvaccinated Pigs, Both with High Levels of MDA at the Age of Vaccination)

**Average daily weight gain.** In total, nine papers reported the ADWG over the entire observation period or enabled the calculation of overall ADGW. The recorded outcomes allowed a total of 21 comparisons, including 11,575 pigs (6791 vaccinated, 4784 unvaccinated). The observation period varied between studies from a minimum of 17 weeks (weeks of age 3 to 20) to a maximum of 25 weeks (weeks of age 1 to 26).

Over all comparisons, vaccinated pigs with high levels of MDA at the time of vaccination gained, on average, 20 g/day more (*p* < 0.001) than their unvaccinated counterparts. Subgroup analyses revealed significant heterogeneity between studies with different types of vaccines; however, the number of comparisons was low for inactivated chimeric PCV1-2 (*n* = 1) and inactivated PCV2 whole virus vaccine *(n* = 2), thereby limiting the validity of the overall conclusions. Outcomes depended on the age at vaccination: pigs vaccinated at 3 weeks (single dose) or at 3 and 6 weeks (two doses) had a significantly higher ADWG (*p* < 0.001). On the other hand, ADWG was not significantly different from unvaccinated pigs if a single vaccine dose was administered <3 weeks of age or >3 weeks of age (*p* > 0.05). The level of MDA (either between 8 and 10 log_2_ or ≥10 log_2_ IPMA titer) had no significant impact on the outcome, whereas the difference in ADWG between vaccinated and unvaccinated pigs was significantly more pronounced in pigs that did not seroconvert. All results are presented in Table 3.

**Mortality.** In total, 11 papers enabled 24 comparisons of the risk of mortality between vaccinated and unvaccinated pigs, including 13,831 pigs (8053 vaccinated, 5778 unvaccinated). The study observation periods varied from a minimum of 12 weeks (weeks of age 4 to 16) to a maximum of 30 weeks (weeks of age 4 to 34).

In the presence of high MDA levels at vaccination, overall mortality in vaccinated pigs was, on average, 34% lower (risk ratio 0.66; *p* < 0.001) than in unvaccinated pigs. Mortality was not affected by vaccine type as heterogeneity between studies with different vaccine types was not significant. On the other hand, the risk of death was dependent on the age at vaccination as pigs vaccinated at 3 weeks or 3 + 6 weeks of age had the highest reduction in mortality, whereas pigs vaccinated at <3 weeks of age had a similar risk of death as unvaccinated pigs (*p* > 0.05). The level of MDA titer at vaccination (either between 8 and 10 log_2_ or ≥10 log_2_ IPMA titer) and the presence or absence of seroconversion had no significant impact on mortality (Table 3).

**Maximum percentage of viremic pigs.** In total, eight papers presented the maximum percentage of viremic pigs (i.e., the highest proportion of viremic pigs measured at any time point during the study observation period) and enabled a total of 15 comparisons, including 1108 pigs (664 vaccinated, 444 unvaccinated). The time point of the highest percentage of viremic pigs varied from ages of 12 to 25 weeks (vaccinated pigs) to ages of 9 to 25 weeks (unvaccinated pigs).

The maximum percentage of viremic pigs was, on average, 63% lower (risk ratio 0.37; *p* < 0.001) in vaccinated pigs compared to their unvaccinated counterparts. The percentage reduction in the highest proportion of viremic pigs ever measured during the observation period was greatest in pigs vaccinated at 3 weeks or >3 weeks of age, and lowest in pigs vaccinated < 3 weeks of age. The heterogeneity between subgroups of different vaccine types, different levels of MDA (either between 8 and 10 log_2_ or ≥10 log_2_ IPMA titer), or the occurrence/absence of seroconversion was not significant. All results are presented in Table 4.

**Maximum log_10_ PCV2 DNA copies.** In total, six papers reported the maximum viral load measured in serum at any time point during the study observation period and enabled a total of 10 comparisons, including 878 pigs (528 vaccinated, 350 unvaccinated). The time point with the highest viral load measured varied from ages of 4 to 23 weeks (vaccinated pigs) to ages of 8 to 23 weeks (unvaccinated pigs).

The maximum viral load was, on average, 0.72 log_10_ PCV2 DNA copies lower (*p* < 0.001) in vaccinated pigs compared to their unvaccinated counterparts. Heterogeneity between studies with different types of vaccines was significant; however, the number of comparisons was low in two subgroups (*n* = 1 and *n* = 2). Maximum viral load depended on the age at vaccination: the greatest percentage reduction was found in pigs vaccinated at 3 weeks or 3 + 6 weeks of age, whereas the maximum titer of log_10_ PCV2 DNA copies was similar to unvaccinated pigs when vaccinated at >3 weeks of age. No data were available for pigs vaccinated at <3 weeks of age. The MDA titer at vaccination (either between 8 and 10 log_2_ or ≥10 log_2_ IPMA titer) had no significant impact on the outcome, whereas the reduction in maximum viral load was higher in groups of pigs that did not seroconvert compared to those that showed seroconversion after vaccination (Table 4).

**Minimum AB titer.** In total, 11 papers enabled the estimation of minimum AB titers in serum, measured at any time point during the study observation period, resulting in a total of 24 comparisons and including 1396 pigs (852 vaccinated, 544 unvaccinated). The time point of minimum AB titer varied from ages of 3 to 34 weeks (vaccinated pigs) and ages of 6 to 38 weeks (unvaccinated pigs).

The minimum AB titer was, on average, 1.46 log_2_ IPMA titer higher in vaccinated pigs than in unvaccinated pigs. Heterogeneity between subgroups of different vaccine types was not significant. Outcomes from subgroup analyses on the age at vaccination revealed significant differences between age groups but were not conclusive as the greatest differences were found in piglets vaccinated at <3 weeks and >3 weeks of age. The MDA titer at vaccination (either between 8 and 10 log_2_ or ≥10 log_2_ IPMA titer) had no significant impact on the outcome, whereas the occurrence of seroconversion led to a higher difference between vaccinated and unvaccinated pigs (Table 5).

**Classic fail-safe N.** The classic fail-safe N test describes the robustness of a significant result against missing papers due to publication or selection bias by estimating how many studies with an effect size of zero could be added to the meta-analysis before the result lost statistical significance. The classic fail-safe N revealed that 2980 studies with no differences in ADWG between vaccinated and unvaccinated pigs were necessary to make the effects non-significant (21 comparisons currently included).

#### 3.2.2. Secondary Analysis (Vaccinated Pigs with High Levels of MDA at Vaccination vs. Vaccinated Pigs with Low to Medium Levels of MDA at Vaccination)

For the production parameters (ADWG and mortality) as well as for the maximum percentage of viremic pigs measured at any time point during the study observation period, outcomes were not different between vaccinated pigs with different levels of MDA at the time of vaccination (either ≥8 log_2_ or <8 log_2_ IPMA titer). In the one comparison which reported the maximum viral load measured at any time point during the study observation period, pigs with a high level of MDA had significantly higher log_10_ PCV2 DNA copies in serum. A summary of all results is presented in Table 6.

## 4. Discussion

The objective of our study was to evaluate the potential interference of MDA with the effectiveness of vaccination in piglets against PCV2. To the best of our knowledge, there are no other literature searches and descriptive or meta-analyses published on this topic.

We aimed to address the following two areas of interest: firstly, if vaccination in pigs with high levels of MDA at the time of vaccination is effective; and secondly, if the effectiveness of vaccination differs in pigs with high levels of MDA at vaccination versus those with low to medium levels.

Our findings confirm the effectiveness of vaccination against PCV2 in the presence of high levels of MDA: vaccination led to a 20 g/day higher ADWG and a 63% reduction in mortality compared to no vaccination when piglets were vaccinated in the face of high MDA. Virological parameters were significantly reduced in vaccinated pigs compared to unvaccinated pigs, as evidenced by the maximum percentage of viremic pigs (minus 63%) and the maximum viral load (minus 0.72 log_10_ PCV2 DNA copies) measured at any time point during the study observation period. A negative correlation between PCV2 viral load and ADWG has already been established [21], thus confirming our findings. ADWG is considered a main driver of profitability for producers [22]; therefore, vaccination of piglets against PCV2 can be regarded as a key attribute to optimize the profitability of swine production even if high levels of MDA, as defined in our study, are present at the time of vaccination.

We also evaluated the impact of covariates on the effectiveness of vaccination against PCV2. From all covariates considered in our analyses, age at vaccination had the most pronounced and consistent impact. Improvements in production parameters (higher ADWG and lower mortality) were greatest in pigs vaccinated once at 3 weeks or twice at 3 and 6 weeks of age. In accordance, virological parameters were most favorable in pigs vaccinated at 3 weeks of age. Piglets vaccinated with a single dose at >3 weeks of age showed no improvement in ADWG. This result, however, should be interpreted with caution, because the majority of the comparisons in this group (6 out of 8) were derived from one single study [14]. Altogether, the least favorable outcomes were observed in studies vaccinating piglets earlier than 3 weeks of age. It is questionable if this finding can be explained by an immaturity of the immune system in these young piglets as for other diseases (e.g., Porcine Reproductive and Respiratory Syndrome and enzootic pneumonia causes by *Mycoplasma hyopneumoniae*) a sufficient immune response to vaccination has been documented even in very young piglets (<1 week of age) [23,24]. Therefore, it can only be speculated if the partly insufficient immune response is specific to PCV2 or to the inactivated subunit vaccines used in the included studies vaccinating piglets < 3 weeks of age. More research would be warranted on this topic. In any case, it is worth emphasizing that the MDA levels considered in our analyses always referred to the titer measured at the time of vaccination as they had to be—*per definitionem*—high at that time, regardless of the age. As such, differences in age at vaccination found in our study were not indirectly related to different MDA levels.

The impact of vaccine type on the effectiveness of vaccination in the presence of high MDA levels cannot be answered conclusively from our analysis. Although heterogeneity between outcomes with different vaccine types was significant for ADWG and the maximum viral load measured, these findings need to be interpreted with caution because of the low number of comparisons included for the inactivated chimeric PCV1-2 (*n* = 1) and inactivated PCV2 whole virus (*n* = 2) vaccines. Additionally, the group of subunit vaccines included not only one but three different products. However, a further split would have led to further small subgroups with even less power.

A minimum MDA titer of ≥8.0 log_2_ IPMA at the age of vaccination was mandatory for inclusion in our analysis because this threshold has been described as a cutoff value below which no interference with vaccination could be expected [6]. However, other authors defined a titer of 10.0 log_2_ IPMA as the threshold for a high MDA level rather than 8.0 log_2_ [5]. Therefore, we aimed to evaluate if the cutoff value might have influenced our results by comparing the outcomes in piglets with MDA levels between 8.0 and 10 log_2_ IPMA titers with those in piglets with MDA levels ≥ 10 log_2_ IPMA titer. For all parameters, heterogeneity between the two subgroups was not significant, thus suggesting no differences between the two groups. However, it must be noted that most studies reported the MDA titer as an average of the entire group of pigs, i.e., the average MDA titer was ≥8.0 log_2_ at the age of vaccination in the groups of vaccinated and unvaccinated piglets, whereas in only one study [7] was the threshold value applied to all piglets, i.e., all piglets had titers ≥8.0 log_2_. Accordingly, it can be reasonably expected that in most studies, the groups with a mean titer of ≥8.0 log_2_ also contained individual piglets with MDA titers < 8.0 log_2_ as well as those with much higher MDA titers. This fact has important consequences: in our overall analyses, comparing vaccinated pigs with unvaccinated pigs with high levels of MDA it might well reflect field conditions, because in cases of high AB levels in the sow (due to vaccination or natural infection), not all piglets will show an identical (high) MDA titer at the age of vaccination. In our subgroup analysis, however, it must be expected that the MDA levels of individual pigs were overlapping between the two comparative groups (MDA levels between 8.0 and 10 log_2_ IPMA titer and MDA levels ≥10 log_2_ IPMA titer), i.e., some piglets in the lower group might have MDA levels higher than 10 log_2_ and some piglets in the high-level group might have levels < 10 log_2_. Accordingly, the outcomes of this subgroup analysis, i.e., the non-significant differences, need to be interpreted with caution. The same limitation applies to our secondary analysis (comparing vaccinated pigs with high levels of MDA versus vaccinated pigs with low to medium levels of MDA at vaccination). In this analysis, however, the potential lack of meaningfulness of non-significant outcomes is less prominent, because the average titer in the low to medium MDA group was much lower, i.e., ≤4.1 log_2_ (with one exception, where it was 7.1 log_2_); thus, smaller overlaps can be rationally assumed. Nevertheless, the non-significant differences between the two groups regarding production parameters and maximum percentage of viremic pigs must still be interpreted with caution. The same applies to the higher viral load in the group of pigs with high levels of MDA, because the outcome is based on one study only.

Seroconversion is the manifestation of a humoral immune response after successful vaccination. It has, however, been shown that the presence of high titers of passively derived maternal ABs can have inhibitory effects on the humoral immune response, potentially preventing the occurrence of seroconversion [5]. Accordingly, only 10 out of 24 studies included in our analyses that allowed the assessment of seroconversion after vaccination showed an increase in the mean AB titer that could be considered as seroconversion. The occurrence or absence of seroconversion after vaccination in the presence of high levels of MDA had an impact on the minimum AB titer measured, because the difference compared to unvaccinated pigs was smaller in those that did not seroconvert. This can be considered a rational finding, because seroconversion is defined as an increase in AB titer after vaccination; thus, it could be expected that the minimum AB titers were higher in pigs that seroconverted compared to those that did not. However, even without seroconversion, the minimum AB titer in vaccinated pigs was still significantly higher compared to unvaccinated pigs, indicative of a serologic response to vaccination even without clear seroconversion. Of course, it cannot be ruled out that—similarly to the limitation outlined for the level of MDA—the group showing no seroconversion on average also included some pigs that actually did seroconvert.

Most interestingly, however, is our finding that outcomes were significantly better (ADWG, maximum viral load), or numerically but not significantly better (mortality, maximum proportion of viremic pigs) in the group of pigs that did not seroconvert after vaccination compared to those that did. We do not have a rational explanation for the better outcomes observed in pigs not showing a vaccine-induced increase in AB titer. However, protection from PCVAD in the absence of a specific serologic response might be due to cellular immunity as all commercially available PCV2 vaccines have been shown to not only induce humoral immune responses, but also cellular immunity [25]. At least, according to our findings, the absence of seroconversion after vaccination in the presence of high levels of MDA should not be assessed as any negative indicator for the effectiveness of vaccination regarding the main production parameters or viremia.

Our analyses have limitations. First of all, in most studies, point estimates and standard deviations or standard errors of the virological and immunological parameters had not been published numerically but had to be derived from line or bar charts using a software specifically developed for digitizing data from scanned graphs (Digitizelt). Additionally, different PCR tests were used in the included studies, which might have had different sensitivities and limits of detection; some studies reported ELISA AB titers, which had to be converted to log_2_ IPMA titers using published conversion rates. Therefore, small deviations from actual values cannot be ruled out. However, because all overall results from primary analyses were highly significant, we believe that this limitation would not change the general statements. Another limitation of the analyses of viral load and minimum AB titer concerns the unit of measurement. All results were log-transformed, which is common practice to allow a suitable graphical presentation of these parameters [26]. Accordingly, the calculated raw mean differences between log-transformed values is not an intuitive measurement, because without re-calculation to non-log transformed titers, it does not reflect the actual difference. However, it is an accepted option to perform meta-analysis on the scale of the log-transformed data [9], and accordingly the calculation of mean differences of log-transformed values has previously been applied in meta-analyses [27].

Another limitation refers to the semi-systematic rather than systematic literature search. A semi-systematic search is less time- and labor-consuming, thus preventing from a substantial time lag between search and ultimate publication of the results, which has been described as a common drawback of systematic reviews [28]. In order to enhance the accuracy of our data search and analyses, a second reviewer partly re-assessed the search results on all three levels. In case of any discrepancy between the two reviewers, results were discussed and corrective measures agreed upon where appropriate. To address any potentially remaining selection bias, we ran the classic fail-safe N statistic for the ADWG, which is a main driver of profitability in swine production, thus being a key factor [22]. The test revealed that 2980 studies with no differences between vaccinated and unvaccinated pigs were needed to make the outcome non-significant. It has been stated that publication bias (or the so-called ‘file drawer problem’) is of no concern if the number of missing studies required to nullify the effects is ≥5k +10 (with k being the number of comparisons currently included) [11]. Using this formula, the tolerance level (X) was highly exceeded (X = 115, calculated missing studies = 2980). Therefore, we are confident that a potential selection bias due to the semi-systematic review is of negligible concern, which likewise applies to a potential publication bias or the exclusion of conference papers.

Last but not least we did not conduct a complete meta-analysis but performed descriptive analysis. Nevertheless, an appropriate test of null hypothesis was performed on all outcomes, and we added statistical components of meta-analysis wherever reasonable (e.g., test of significant heterogeneity between subgroups, classic fail-safe N). Thus, we are confident that our results are statistically sound and meaningful.

## 5. Conclusions

The vaccination of piglets in the presence of high levels of MDA is effective with respect to production parameters (ADWG and mortality), viremia (maximum percentage of PCR-positive pigs, maximum viral load), and immunological parameters (minimum AB titers). Our study indicates that vaccination at 3 weeks of age is most advantageous regarding production parameters and viremia if high titers of MDA are expected. The potential absence of seroconversion after vaccination in the presence of high levels of MDA had no negative impact on production parameters (ADWG and mortality) or viremia.

## Figures and Tables

**Table 1:** Characteristics of studies identified through semi-systematic literature search and included for the evaluation of interference of MDA with the effectiveness of vaccination against PCV2.

Study				Study Characteristics				Number of Comparisons				
Reference	Ref.	Separate Groups (If Applicable)	Country	Type of Vaccine	Age at Vacc. (Weeks)	MDA Level (All ≥ 8 log_2_)	Seroconversion	ADWG	Mortality	Max. % Viremic	Max. Viral Load	Min. AB Titer
Csank et al., 2013	[12]		Slovak Rep.	Inact. subunit ORF2	2	≥10 log_2_	Yes	0	0	0	0	1
Feng et al., 2016	[7]		Spain	Inact. subunit ORF2	3	≥10 log_2_	No	1	1	1	1	1
Figueras-Gourgues et al., 2019	[4]		Germany, UK, France	Inact. subunit ORF2	3	<10 log_2_	n.r. *	1	1	0	0	0
Fraile et al., 2012	[13]	Sows vacc.	Spain	Inact. subunit ORF2	4	≥10 log_2_	Yes	1	1	1	1	1
		Sows unvacc.	Spain	Inact. subunit ORF2	4	<10 log_2_	n.a. ^#^	1	1	1	1	1
Fraile et al., 2012	[5]	Farm A	Spain	Inact. whole virus	3	<10 log_2_	Yes	1	1	1	1	1
Haake et al., 2014	[8]	Study 1a	Europe	Inact. subunit ORF2	1	≥10 log_2_	No	1	1	1	0	1
		Study 1b	Europe	Inact. subunit ORF2	3	≥10 log_2_	No	1	1	1	0	1
		Study 2a	Europe	Inact. subunit ORF2	1	≥10 log_2_	No	1	1	1	0	1
		Study 2b	Europe	Inact. subunit ORF2	3	<10 log_2_	Yes	1	1	1	0	1
Martelli et al., 2016	[14]	Rep. 1-A	Italy	Inact. subunit ORF2	4	≥10 log_2_	Yes	1	1	0	0	1
		Rep. 2-A	Italy	Inact. subunit ORF2	4	≥10 log_2_	No	1	1	0	0	1
		Rep. 2-B	Italy	Inact. subunit ORF2	6	≥10 log_2_	Yes	1	1	0	0	1
		Rep. 2-C	Italy	Inact. subunit ORF2	8	<10 log_2_	Yes	1	1	0	0	1
		Rep. 3-A	Italy	Inact. subunit ORF2	4	≥10 log_2_	No	1	1	0	0	1
		Rep. 3-B	Italy	Inact. subunit ORF2	6	≥10 log_2_	No	1	1	1	0	1
Paphavasit et al., 2009	[15]		Thailand	Inact. chimeric PCV1-2	4	≥10 log_2_	Yes	0	1	1	0	1
Stevancevic et al., 2014	[16]		Serbia	Inact. subunit ORF2	2	<10 log_2_	Yes	0	1	0	0	1
				Inact. subunit ORF2	3	<10 log_2_	Yes	0	1	0	0	1
Tassis et al., 2017	[17]		Greece	Inact. subunit ORF2	3	≥10 log_2_	No	1	1	0	1	0
Tzika et al., 2015	[18]		Greece	Inact. subunit ORF2	3	<10 log_2_	No	1	1	1	0	1
Vagas-Bermudez et al., 2018	[19]		Colombia	Inact. subunit ORF2	3	≥10 log_2_	No	0	0	0	1	1
Villa-Mancera et al., 2016	[20]		Mexico	Inact. chimeric PCV1-2	3	≥10 log_2_	No	1	1	1	1	1
			Mexico	Inact. PCV2 whole virus	3	≥10 log_2_	No	1	1	1	1	1
			Mexico	Inact. subunit ORF2	3	≥10 log_2_	No	1	1	1	1	1
			Mexico	Inact. subunit ORF2	3+ 6	≥10 log_2_	No	1	1	1	1	1

MDA, maternally derived antibodies; PCV2, porcine circovirus type 2; Ref., reference; vacc., vaccination/vaccinated; ADWG, average daily weight gain; Max., maximum; Min., minimum; AB, antibody; Inact., inactivated; ORF2, open reading frame 2; * n.r., not reported; ^#^ n.a., not assessable (as seroconversion also occurred in unvaccinated pigs, indicating natural infection).

**Table 2:** Characteristics of the subgroup of studies additional to the group of pigs vaccinated against PCV2 in the presence of high levels of MDA (≥8 log_2_ IPMA antibodies) which also reported a subgroup of pigs with low to medium levels of MDA (<8 log_2_ IPMA antibodies) at the age of vaccination.

Study				Study Characteristics				Number of Comparisons			
Reference	Ref.	Study Location(s)	Type of Vaccine	Age at Vacc. (High Level Group)	Age at Vacc. (Low to Medium Level Group)	MDA Level (High Level Group)	MDA Level (Low to Medium Level Group)	ADWG	Mortality	Max. % Viremic	Max. Viral Load
Feng et al., 2016	[7]	Spain	Inact. subunit ORF2	3 weeks	3 weeks	≥10 log_2_	3.8 log_2_	1	1	1	1
Fraile et al., 2012	[5]	Spain	Inact. PCV2 whole virus	3 weeks	3 weeks	≥8 log_2_	7.1 log_2_	1	1	1	0
Martelli et al., 2016 (4 comparisons)	[14]	Italy	Inact. subunit ORF2	4–6 weeks	6–8 weeks	≥10 log_2_	≤4.1 log_2_	4	4	0	0

MDA, maternally derived antibodies; PCV2, porcine circovirus type 2; Ref., reference; vacc., vaccination; ADWG, average daily weight gain; Max., maximum; Inact., inactivated; ORF2, open reading frame 2.

**Table 3:** Differences in production parameters (average daily weight gain and mortality) between vaccinated and unvaccinated pigs with high levels of MDA at the age of vaccination against PCV2.

		ADWG (g/day)			Mortality		
		n	Mean Difference (95% CI)	Significance (*p*-Value)	n	Risk Ratio (95% CI)	Significance (*p*-Value)
**Overall Analysis**		21	+20.32 (14.97; 25.68)	<0.001	24	0.66 (0.57; 0.77)	<0.001
**Subgroup Analyses**		**n**	**Mean Difference**	**Heterogeneity between Subgroups** ***(p*** **-Value)**	**n**	**Risk Ratio**	**Heterogeneity between Subgroups** ***(p*** **-Value)**
Vaccine used in piglets	Inact. chimeric PCV1-2	1	+32.00	<0.001	2	0.63	0.492
	Inact. subunit ORF2	18	+14.74		20	0.66	
	Inact. PCV2 whole virus	2	+24.95		2	0.51	
Age at vaccination (weeks)	<3	2	−0.78	0.001	3	1.14	0.030
	3	10	+24.88		11	0.61	
	>3	8	−6.31		9	0.77	
	3 + 6 *	1	+32.00		1	0.41	
MDA titer (IPMA) at vaccination	≥8 and <10 log_2_	6	+13.28	0.162	8	0.67	0.822
	≥10 log_2_	15	+24.41		16	0.65	
Seroconversion after vaccination	Yes	6 ^#^	+3.18	0.001	9 ^#^	0.82	0.175
	No	13 ^#^	+27.41		13 ^#^	0.63	

* Vaccinated twice: at 3 and at 6 weeks of age; ^#^ the sum is smaller than the total number of comparisons, because vaccine-induced seroconversion was not assessable in studies where an increase in antibody titer simultaneously occurred in unvaccinated pigs, being indicative of natural infection. MDA, maternally derived antibodies; PCV2, porcine circovirus type 2; ADWG, average daily weight gain; CI, confidence interval; n, number of comparisons; Inact., inactivated; ORF2, open reading frame 2.

**Table 4:** Differences in virological parameters (maximum percentage of viremic pigs and maximum PCV2 DNA copies in serum at any time during the study observation period) between vaccinated and unvaccinated pigs with high levels of MDA at the age of vaccination against PCV2.

		Max. % of Viremic Pigs			Max. PCV2 DNA Copies (log_10_)		
		n	Risk Ratio (95% CI)	Significance (*p*-Value)	n	Mean Difference (95% CI)	Significance (*p*-Value)
**Overall Analysis**		15	0.37 (0.27; 0.53)	<0.001	10	–0.72 (–0.90; –0.54)	<0.001
**Subgroup Analyses**		**n**	**Risk Ratio**	**Heterogeneity between Subgroups** ***(p*** **-Value)**	**n**	**Mean Difference**	**Heterogeneity between Subgroups** ***(p*** **-Value)**
Vaccine used in piglets	Inact. chimeric PCV1-2	2	0.32	0.967	1	–1.03	0.004
	Inact. subunit ORF2	11	0.37		7	–0.59	
	Inact. PCV2 whole virus	2	0.39		2	–0.86	
Age at vaccination (weeks)	<3	2	0.67	0.026	0		0.012
	3	8	0.37		7	–0.97	
	>3	4	0.22		2	+0.03	
	3 + 6 *	1	0.58		1	–0.87	
MDA titer (IPMA) at vaccination	≥8 and <10 log_2_	4	0.29	0.164	2	–0.10	0.193
	≥10 log_2_	11	0.41		8	–0.89	
Seroconversion after vaccination	Yes	4 ^#^	0.28	0.065	2 ^#^	–0.45	0.003
	No	10 ^#^	0.45		7 ^#^	–0.93	

* Vaccinated twice at 3 and 6 weeks of age; ^#^ the sum is smaller than total number of comparisons, because vaccine-induced seroconversion was not assessable in studies where an increase in antibody titer simultaneously occurred in unvaccinated pigs, being indicative of natural infection. MDA, maternally derived antibodies; PCV2, porcine circovirus type 2; Max., maximum; n, number of comparisons; CI, confidence interval; Inact., inactivated; ORF2, open reading frame 2.

**Table 5:** Differences in serological parameters (minimum AB titer in serum measured at any time point during the study observation period) between vaccinated and unvaccinated pigs with high levels of MDA at the age of vaccination against PCV2.

		Min. PCV2 AB Titer (log_2_)		
		n	Mean Difference (95% CI)	Significance (*p*-Value)
**Overall Analysis**		24	+1.46 (1.19; 1.73)	<0.001
**Subgroup Analyses**		**n**	**Mean Difference**	**Heterogeneity between Subgroups** ***(p*** **-Value)**
Vaccine used in piglets	Inact. chimeric PCV1-2	2	+0.95	0.091
	Inact. subunit ORF2	10	+1.76	
	Inact. PCV2 whole virus	2	+0.48	
Age at vaccination (weeks)	<3	4	+1.65	<0.001
	3	10	+0.49	
	>3	9	+2.42	
	3 + 6 *	1	+0.06	
MDA titer (IPMA) at vaccination	≥8 and <10 log_2_	7	+2.13	0.194
	≥10 log_2_	17	+1.31	
Seroconversion after vaccination	Yes	10 ^#^	+2.51	0.003
	No	13 ^#^	+0.85	

* Vaccinated twice at 3 and 6 weeks of age; ^#^ the sum is smaller than the total number of comparisons, because vaccine-induced seroconversion was not assessable in studies where an increase in antibody titer simultaneously occurred in unvaccinated pigs, being indicative of natural infection. MDA, maternally derived antibodies; PCV2, porcine circovirus type 2; AB, antibody; Min., minimum; n, number of comparisons; CI, confidence interval; Inact., inactivated; ORF2, open reading frame 2.

**Table 6:** Comparison of outcomes of production (ADWG and mortality) and virological parameters (maximum percentage of viremic pigs and maximum viral load in serum at any time point during the study observation period) between vaccinated pigs with high versus low to medium levels of MDA at the age of vaccination against PCV2.

Parameter	n	Analyses		
		Effect Size	Result (95% CI)	Significance (*p*-Value)
ADWG (g/d)	6	Mean Difference	+2.24 (−37.0–41.5)	0.911
Mortality	6	Risk Ratio	0.77 (0.53–1.13)	0.179
Max. % viremic pigs	2	Risk Ratio	1.14 (0.60–2.15)	0.694
Max. viral load (log_10_ PCV2 DNA copies)	1	Mean Difference	+0.45 (0.10–0.80)	0.011

MDA, maternally derived antibodies; PCV2, porcine circovirus type 2; CI, confidence interval; ADWG, average daily weight gain; Max, maximum.

## Data Availability

Data are available from the corresponding author upon request.
